# Phospholipases and Reactive Oxygen Species Derived Lipid Biomarkers in Healthy and Diseased Humans and Animals – A Focus on Lysophosphatidylcholine

**DOI:** 10.3389/fphys.2021.732319

**Published:** 2021-11-10

**Authors:** Kathrin M. Engel, Jürgen Schiller, Christina E. Galuska, Beate Fuchs

**Affiliations:** ^1^Faculty of Medicine, Institute of Medical Physics and Biophysics, University of Leipzig, Leipzig, Germany; ^2^Core Facility Metabolomics, Research Institute for Farm Animal Biology (FBN), Dummerstorf, Germany

**Keywords:** disease markers, inflammation, lysophospholipids, lysophosphatidylcholine, phospholipids, phospholipase, reactive oxygen species, disease marker

## Abstract

Phospholipids (PL) are converted into lipid biomarkers by the action of phospholipases and reactive oxygen species (ROS), which are activated or released under certain physiological and pathophysiological conditions. Therefore, the *in vivo* concentration of such lipid biomarkers [e.g., lysophospholipids (LPLs)] is altered in humans and animals under different conditions such as inflammation, stress, medication, and nutrition. LPLs are particularly interesting because they are known to possess pro- and anti-inflammatory properties and may be generated by two different pathways: either by the influence of phospholipase A_2_ or by different reactive oxygen species that are generated in significant amounts under inflammatory conditions. Both lead to the cleavage of unsaturated acyl residues. This review provides a short summary of the mechanisms by which lipid biomarkers are generated under *in vitro* and *in vivo* conditions. The focus will be on lysophosphatidylcholine (LPC) because usually, this is the LPL species which occurs in the highest concentration and is, thus, easily detectable by chromatographic and spectroscopic methods. Finally, the effects of lipid biomarkers as signaling molecules and their roles in different human and animal pathologies such as infertility, cancer, atherosclerosis, and aging will be shortly discussed.

## Introduction – Phospholipids as Educts of Relevant Physiological Molecules

Phospholipids (PL) constitute an important class of biomolecules, among which glycerophospholipids (GPL) are of high relevance (Law et al., 2019). All GPLs consist of a glycerol backbone, where two hydroxyl groups are esterified with two (often varying) fatty acids. The third hydroxyl group is esterified with phosphoric acid. The resulting molecule is termed “phosphatidic acid” (PA). *Via* ester condensation with different alcohols such as choline or ethanolamine, phosphatidylcholine (PC), and phosphatidylethanolamine (PE) are generated. These compounds represent the most abundant zwitterionic GPL in mammalian membranes.

**FIGURE 1:**
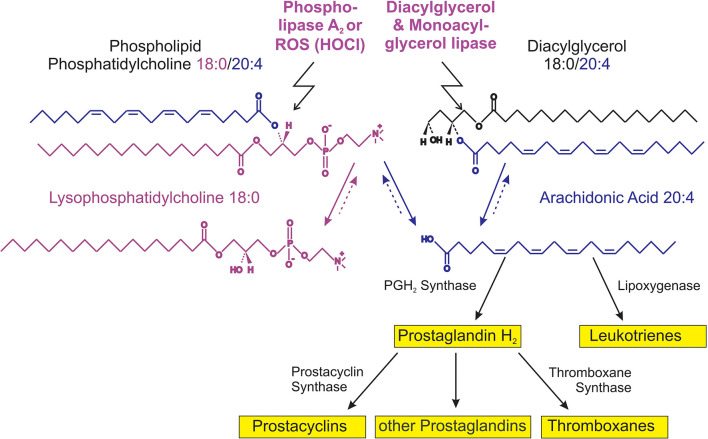
Survey of lysophosphatidylcholine (LPC) metabolism.

The majority of PLs occurring under *in vivo* conditions are characterized by a saturated fatty acyl residue in *sn*-1 position, while the second fatty acyl residue is often mono- (e.g., oleic acid), di- (e.g., linoleic acid) or even higher unsaturated (e.g., arachidonic or docosahexaenoic acid, which contain four or six double bonds, respectively). GPLs are converted into lysophospholipids (LPLs) by the action of phospholipases. The reactions catalyzed by these enzymes are illustrated in Figure 1. The released free fatty acids have considerable physiological significance: highly unsaturated fatty acids such as arachidonic acid (C20:4) are very sensitive to oxidation and its metabolic (oxidation) products such as thromboxanes, prostaglandins, or leukotrienes have a considerable biological impact (Johnson et al., 2020).

Both, the LPL as well as the free fatty acid are considered as important molecules with potential messenger functions and destabilize cellular membranes due to their detergent-like character (Hu et al., 2007).

## Generation of Lysophospholipid Under the Influence of Phospholipases and Reactive Oxygen Species

Phospholipase “A” (PLA) is represented by a group of enzymes that catalyze the hydrolysis of one fatty acyl residue from the glycerol backbone of a PL. By this reaction, a free fatty acid is released and the corresponding LPL is left in the membrane (Peng et al., 2021; Figure 1).

The cleavage of phosphatidylcholine (PC) by PLA_2_ yields lysophosphatidylcholine (LPC) and free fatty acids, e.g., arachidonic acid, the educt of eicosanoids. The hydrolysis of diacylglycerol (DAG) by DAG lipase at the *sn-*1 position results in 2-arachidonoylglycerol (2-AG) followed by a monoacylglycerol lipase (MAGL)-dependent hydrolysis of 2-AG to generate arachidonic acid and further eicosanoid pathway metabolites. Please note that more degradation products such as lysophosphatidic acid (LPA) (Hosogaya et al., 2008) (LPA) and glycerophosphocholine (GPC) can be produced by other phospholipases as well as reconversion by acyltransferases can take place (Brindley and Bräuer, 2010).

Based on the stereospecificity of the reaction, PLA_1_ and PLA_2_ activities can be differentiated. PLA_1_ enzymes generally play a minor role than PLA_2_ (Richmond and Smith, 2011), although there is increasing evidence that PLA_1_ activity is underestimated regarding the generation of lysophosphatidylserine (LPS) (Iwata et al., 2021). The prevailing opinion is that LPLs are generated under *in vivo* conditions by the release and/or activation of PLA_2_ that is particularly present in neutrophilic granulocytes, important cellular mediators of inflammation. However, neutrophils do not only secrete PLA_2_, but are also capable of generating reactive oxygen species (ROS; Pérez-Figueroa et al., 2021). All ROS are derived from atmospheric air oxygen, which is converted in a set of reactions into H_2_O_2_. This is the precursor for the generation of further, much more reactive species, for instance, hydroxyl radicals (HO^•^). Despite the high reactivity of HO^•^, that reacts diffusion-controlled with nearly all organic molecules, another ROS seems to be responsible for the increased levels of LPC under pathological conditions: hypochlorous acid (HOCl; Schröter and Schiller, 2016). HOCl is generated under *in vivo* conditions from H_2_O_2_ and Cl^–^ ions under catalysis of myeloperoxidase (MPO; Kargapolova et al., 2021):


$$H_{2}\,O_{2}+{\mathrm{Cl}}^{-}\to \mathrm{HOCl}+{\mathrm{HO}}^{-}$$

Myeloperoxidase is nearly exclusively found in neutrophils, where it amounts to approximately 5% of the total protein content (Schröter and Schiller, 2016). As the number of neutrophilic granulocytes increases massively under inflammatory conditions, the roles of MPO and its products are obvious (Arnhold, 2020). *In vitro*, it could be shown that LPC is also generated from isolated PC by the reaction with HOCl (Arnhold et al., 2002).

## Methods of Lysophospholipid Determinations

Methods of LPL determinations often rely on, for instance, UV-, fluorescence, or ESR Spectroscopy, radioactivity or capillary electrophoresis. These methods detect mainly the related phospholipase activities, need prior labeling and/or do not give detailed structural information of the generated LPL. We will focus here on NMR (particularly ^31^P and ^1^H NMR), chromatography (mainly HPLC and TLC) and mass spectrometry as these methods overcome many disadvantages of the methods mentioned above – in particular, they do not require any specific labels. The advantages and drawbacks as well as details of the individual techniques are compared in Table 1.

**TABLE 1:** Survey of selected techniques of lysophospholipid (LPL) analysis.

	Principle	Advantages	Drawbacks	Remarks
High-performance liquid chromatography (HPLC)	Separation on a “stationary phase” under high pressure by elution with solvents of different polarities; “reversed” phase is more common than normal phase.	Can be standardized; protocols are available for many analytes; coupling with MS is well established - although detection by UV or light scattering is still widely used; preparative HPLC is possible.	Requires experienced operators; detection of saturated lipids (lack of UV absorptions) is difficult; post-column derivatization is time-consuming.	Routine method in many laboratories; “fine-tuning” of the mobile phase to the relevant sample is normally required; a timely review with the focus on liposomes and the detection of LPC as impurity is available in de Magalhães Benedetti et al. (2020).
Thin-layer chromatography (TLC)	Separation on a stationary phase (normally silica gel) due to polarity differences of the analytes; different modifications/polarities of the stationary phase are commercially available	Inexpensive and fast; many samples can be simultaneously analyzed; stainings with different properties (non-covalent, covalent binding, UV, fluorenscence detection) can be used for detection.	Oxidation (of unsaturated LPL species) may occur during the run; resolving LPL with different acyl residues is difficult; less sensitive compared to MS.	Often used as initial method if a complex lipid mixture has to be analyzed in detail; TLC-based lipid analysis is still common (Adibi et al., 2018).
ESI MS	Ions are generated from charged droplets.	“very soft” ionization method; little analyte fragmentation; quantification possible in the presence of a suitable internal standard.	Ion suppression may occur; strongly affected by sample impurities as well as the composition of the solvent.	ESI MS is already in clinical use since metabolite determination by MS is often cheaper than other methods (Liebisch et al., 2002).
MALDI MS	Ions are generated by laser irradiation of a matrix/analyte cocrystal	“soft” ionization method; little analyte fragmentation; very little sample pretreatment and purification required.	Ion suppression may occur; obtaining quantitative data is difficult.	PC/LPC ratios are often given which can be calculated without internal standards (Bresler et al., 2011).
^1^H NMR	Differences in electron densities lead to different chemical shifts of the observed nucleus within a given compound	Basically all lipids are detectable; isomers can be differentiated without the need of previous separation; spectra exhibit quantitative information.	Analyses of mixtures lead to complex spectra; need of deuterated solvents; expensive equipment; differences in the fatty acyl compositions can hardly be resolved.	One characteristic functional group (the quaternary ammonia group) is used as the sensor to detect the different classes; high magnetic field strengths are required to resolve PC, LPC and SM (same headgroups) (Soininen et al., 2007).
^31^P NMR	The different chemical environment renders each P atom a characteristic chemical shift.	Direct absolute quantitation is possible; isomeric lipids can be differentiated.	Limited sensitivity (order of magnitude less than MS); requires high amounts of sample; expensive equipment.	Detergents or solvent mixtures have to be used in order to suppress the aggregation of phospholipids; acyl migration may falsify the results (Sugasini and Subbaiah, 2017); has been recently reviewed: (Li et al., 2017).

*The advantages and drawbacks of the different methods are summarized. Note that chromatographic separation is often combined with MS detection. A review dealing with selected methods is available in Helmerich and Koehler (2003).*

It is important to note, that LPC can be generated from PC even under *in vitro* conditions. That is, even solutions of PC that are meant to be pure still often contain small amounts of LPC and the LPC moiety increases during storage (Hernandez-Caselles et al., 1990). Of course, the amount of detected LPC is also influenced by the applied extraction protocol because LPC is much more polar than other lipids. MALDI MS has the considerable advantage that LPC may also be determined from native samples, i.e., without the necessity of sample extraction that may result in the LPL loss. These aspects were recently discussed by Ditz and Fuchs (2018) and have already been comprehensively reviewed by Petković et al. (2005).

## Lysophospholipid as Inflammation and Disease Markers

Lysophospholipid (LPL) such as LPC, LPA, sphingosine-1-phosphate (S1P), LPS (Kurano et al., 2015), and lysophosphatidylinositol (LPI) have pronounced effects on diverse cell lines and the immunological effects induced by these compounds have already been reviewed (Xu et al., 2013). For some LPL, such as LPI and LPS signaling *via* G protein-coupled receptors (GPCR) has been described and was comprehensively reviewed by Tan et al. (2020). Even though the possibility of GPCR-mediated function of LPC has been pointed out, there is currently no GPCR for LPC binding known (Makide et al., 2014). Despite many studies revealed pro-inflammatory effects of LPL – such as LPC as key marker that is positively associated with cardiovascular and neurodegenerative diseases – there are also research articles demonstrating anti-inflammatory effects of these compounds, making findings controversial (Knuplez and Marsche, 2020). This controversial behavior can be explained by the *in vivo* generation of two different compounds: LPL and free fatty acids. In inflammation, the released free fatty acid is often arachidonic acid that is readily converted into compounds with immunomodulatory effects. Furthermore, LPC is a precursor of extracellular LPA, an important lipid mediator and modulator of neuronal function (Brindley and Bräuer, 2010). LPA is produced from LPC by autotaxin and is therefore a direct executioner of LPC (Hosogaya et al., 2008; Yatomi et al., 2018). However, the concentration of LPA in body fluids and tissues is always much lower (normally about two orders of magnitude) compared to LPC. In a nutshell, both, the generation of LPL and the formation of arachidonate-derived metabolites must be inhibited for pharmacological effects (Sun et al., 2014).

There is one interesting study that illustrates the potential “marker quality” of LPC: it was shown that horse sera with a high LPC content are not useful for cell experiments because they have deleterious effects on cell growth. This suits the finding that horse sera with moderate amounts of LPC were also characterized by low levels of inflammatory eicosanoids (Ditz et al., 2019).

### Lysophosphatidylcholine Under Inflammation and Stress

The concentration of LPC increases under inflammatory conditions. This has been shown, for instance, in joint fluids from rheumatoid arthritis patients (Fuchs et al., 2005) or atherosclerosis patients (Matsumoto et al., 2007). Under certain conditions, however, the opposite was observed: plasma LPC showed an inverse relationship with cardiovascular diseases (Ganna et al., 2014). In other studies, the LPC/PC ratio in plasma as well as cerebrospinal fluid from patients with Alzheimer’s disease decreased (Lin et al., 2017). Unfortunately, all these studies were methodologically different and, thus, the comparability is poor.

Lysophosphatidylcholine and LPE levels rise considerably in human inflammatory liver tissue (Schober et al., 2009). Particularly, LPC could serve as a biomarker in fatty liver disease. However, it is known that also a metabolically healthy fatty liver exists (Stefan et al., 2019). The comparison of molecule pattern of plasma from insulin-sensitive and insulin-resistant human subjects have shown that particularly LPCs are capable to distinguish benign and malignant non-alcoholic fatty liver (Lehmann et al., 2013) and mechanisms involved in this process, e.g., pro-inflammatory signaling (*via* regulation of LPC acyltransferase) were discussed. Similarly, in studies with non-alcoholic steatohepatitis in mice major reductions in LPC 16:0, 18:0 and 18:1 were identified (Tanaka et al., 2012).

It could also be shown that LPC enhances the generation of superoxide anion radicals (O_2_^•–^) as well as H_2_O_2_ (Englberger et al., 1987), i.e., LPC may trigger the generation of even stronger ROS such as HO^•^ or HOCl. Similar observations were made for lymphocytes, at which the presence of LPC led to an increased number of apoptotic cells. However, it is not known whether these studies are relevant under *in vivo* conditions. *In vivo*, there is a huge amount of proteins with high affinity for LPC, e.g., albumin and lipoproteins. Thus, the amount of physiologically available LPC may vary considerably. Anyway, LPC normally does not accumulate in the body because different mechanisms limit the elevation of LPC concentrations: (I) the re-acylation of LPC to PC, (II) the degradation of LPC to GPC by the cleavage of the fatty acyl residue in the *sn*-1 position by lysophospholipases. GPC lacks both acyl residues and is, thus, soluble in water. An overview of the PC- and LPC-related pathways was provided by Fuchs et al. (2012).

Despite the many open questions, targeting LPC and its metabolic pathways might be a prospective treatment strategy of inflammatory diseases (Liu et al., 2020).

### Lysophospholipid in Fertility and Infertility

Male gametes are perhaps the most important cells at which LPL are known to have significant impact. The related process is called “capacitation”: mammalian spermatozoa undergo a variety of physiological events, which make them ready for fertilization, i.e., the fusion between the sperm and the female oocyte.

Ye (2008) reviewed the physiological functions of LPL (particularly LPA and S1P) in reproduction as well as potential pathological side effects. LPC has been shown to have an impact on human corporal smooth muscle cells, and therefore, might lead to an impaired penile function through TPR channels (So et al., 2005). Furthermore, it is known since many years that LPC is capable of triggering sperm acrosomal exocytosis, an important event that primes spermatozoa for successful fertilization (Ehrenwald et al., 1988). Roldan and Shi (2007) examined the action of PLA_2_ and its role for successful fertilization. There are two obvious functions of the generated LPL: on the one hand, they represent second messengers for cellular signaling. On the other hand, they act as detergents and destabilize the membranes by changing their biophysical properties. Despite the obvious physiological role of PLA_2_ activation, spermatozoa avoid premature destabilization and, thus, a carefully controlled equilibrium between LPL generation and its reacylation into the corresponding PL must exist. Deviations from normal conditions may otherwise lead to pathological situations, so that an enhanced sperm LPL concentration is presumably indicative of a reduced fertilizing potential. There is evidence that the LPC content reciprocally correlates with the sperm quality of human sperm (Roldan and Shi, 2007). Using magnetic assisted cell sorting (MACS) to separate intact and apoptotic (annexin V positive) spermatozoa, it was shown that the LPC content is much higher in the apoptotic sperm (Glander et al., 2002). Although it is not yet clear whether the observed effect is triggered by an enhanced generation of ROS or an elevated PLA_2_ activity, there are many indications that oxidative stress has a pronounced impact on sperm physiology (Takeshima et al., 2020). For instance, sperm from extremely obese men are characterized by an enhanced LPC content. This correlates with a reduced fertilizing capacity of these men (Pyttel et al., 2012).

Thus, LPC might represent an interesting “marker” molecule in fertility that definitely is worth of further investigations because it has a considerable advantage: in comparison to proteins, LPC is a non-specific marker that could be useful for human as well as animal spermatozoa (Nimptsch et al., 2014).

### Lysophosphatidylcholine and Aging

It is known that lipids play a role in lifespan regulation and age-related diseases (de Diego et al., 2019). Very recently, the use of lipidomics to age-related musculoskeletal disorders was reviewed (Mo et al., 2021). Siddharth et al. (2017) monitored changes of serum lipids in aged mice with a sarcopenic phenotype and found that levels of LPC 20:5 and LPC 20:3 were reduced – although an explanation of the occurrence of these unusual fatty acids was not provided. In humans blood LPC levels tend to decrease with age: low plasma levels of LPC are associated with impaired mitochondrial oxidative capacity in adults (Semba et al., 2019). Lower levels of LPC 18:2 were shown to be predictive of memory impairment and enable the prediction of a greater decline of gait speed in the elderly (Gonzalez-Freire et al., 2019).

Patients with cancer, an age-related disease, exhibit a decreased LPC concentration in plasma (Taylor et al., 2007). Similarly, Kim et al. (2014) found that the LPC 24:0 levels in aged mice were lower compared to young mice. Although no explanation of the occurrence of this less common organic residue (lignoceric acid) is provided, the authors conclude that the ratios of the individual metabolites PC and LPC could serve as potential biomarkers for aging and age-related diseases. In contrast, there are also studies showing that during aging the levels of LPC increase – in particular in patients suffering from Alzheimer’s disease (Dorninger et al., 2018). Although not yet carefully studied, LPC seems to enhance oxidative stress *via* the 5-lipoxygenase pathway in rat aorta during aging (Zou et al., 2009). This might foster further pharmacological studies because lipoxygenase is one interesting target for many drugs.

### Lysophosphatidylcholine Under Medication, Nutrition, and Pharmacological Aspects

The normal concentration of LPC in plasma is significant (about 200–300 μM) – with most LPC bound to albumin and lipoproteins (Kishimoto et al., 2002). Anyway, LPC-treatment of mice induces enhanced phagocytic activity of macrophages (Lee et al., 2018). Intracutaneous injection of LPC in healthy volunteers similarly elicited acute inflammation with the accumulation of T lymphocytes, monocytes, and neutrophils (Murugesan et al., 2003). Direct pro-inflammatory and atherogenic effects of LPC have become apparent over the past 30 years. Nowadays, there is increasing evidence that LPC has also anti-inflammatory properties, making its profile more complex than initially assumed (Schilke et al., 2020). These controversial effects of LPC are presumably caused by differences in length and/or the saturation state of the fatty acyl chain (Tan et al., 2020). Additionally, different functional effects of LPC may also be due to different moieties of the free and the albumin-bound LPC (Mehta, 2005).

Dietary polar lipids are relevant for the cognitive development and are distributed throughout the body by lipoproteins (Zheng et al., 2019). Lipids play an unequivocal active part in the acceptability, flavor, and perception of foods and may be beneficial for health – or lead to various pathologies (Meynier and Genot, 2017). Finally, LPC is used in animal nutrition to improve animal performance, i.e., to favor the digestion of food and the release of nutrients from the diet (Wealleans et al., 2020).

As LPC concentration is elevated in many pathologies, different attempts were undertaken to decrease the LPC concentration *in vivo*. Because of the obvious contribution of PLA_2_, this has raised interest for pharmacologically-active substances capable of inhibiting PLA_2_ activity (Yalagala et al., 2019; Frangieh et al., 2021). However, PLA_2_ activation does not only result in LPC generation but also in arachidonate-derived free radical intermediates (Sun et al., 2014) and further ROS. Therefore, a single drug molecule with both – anti-oxidative and PLA_2_ inhibitory activity – would be useful since it could inhibit PLA_2_ activity and simultaneously scavenge free radicals and lipid peroxides which are released during C20:4 metabolism.

## Concluding Remarks

Lysophospholipids represent lipid molecules with a Janus-faced character: on the one hand, LPL are important signaling molecules that are required for physiological processes, such as successful fertilization or proper memory function. On the other hand, their *in vivo* concentrations has to be carefully controlled, e.g., a disturbance of the equilibrium between LPC generation and re-acylation can lead to severe pathological conditions. The focus of this review is on LPC and this is also true for previous studies. This is, at least partially, caused by the fact that this LPL can be most sensitively detected. This can be achieved with low resolution mass spectrometers. Furthermore, LPC is a comparably stable compound, that does not react with other compounds that is again an excellent property for its successful determination. It remains to be elucidated whether and which LPL are useful and reliable biomarkers of (inflammatory) diseases. Contradicting effects of LPC observed in experimental models and patient samples could be due to differences in saturation and/or length of the fatty acyl chain.

## Author Contributions

JS wrote chapter 3 including Table 1. KE wrote chapter 4.2. BF wrote chapter 2 including Figure 1 and chapter 4.3. CG wrote chapter 4.4. All authors contributed to the other chapters, abstract, introduction, and conclusions.

## Conflict of Interest

The authors declare that the research was conducted in the absence of any commercial or financial relationships that could be construed as a potential conflict of interest.

## Publisher’s Note

All claims expressed in this article are solely those of the authors and do not necessarily represent those of their affiliated organizations, or those of the publisher, the editors and the reviewers. Any product that may be evaluated in this article, or claim that may be made by its manufacturer, is not guaranteed or endorsed by the publisher.
